# Effectiveness and safety of liposomal bupivacaine in autologous breast reconstruction: a systematic review

**DOI:** 10.3389/fmed.2026.1805974

**Published:** 2026-05-07

**Authors:** Waiel A. Daghistani

**Affiliations:** Department of Anesthesia and Surgery, College of Medicine, Imam Mohammad Ibn Saud Islamic University (IMSIU), Riyadh, Saudi Arabia

**Keywords:** autologous breast reconstruction, breast, liposomal bupivacaine, postoperative pain management, regional anesthesia

## Abstract

**Background:**

Autologous breast reconstruction (ABR), particularly using abdominally based free flaps such as the deep inferior epigastric perforator (DIEP) flap, is associated with significant postoperative pain. Liposomal bupivacaine, a long-acting local anesthetic, has been increasingly incorporated into multimodal analgesia protocols; however, its effectiveness in improving postoperative outcomes remains uncertain.

**Objective:**

To systematically evaluate the effectiveness and safety of liposomal bupivacaine in reducing opioid consumption and improving recovery outcomes in patients undergoing autologous breast reconstruction.

**Methods:**

A systematic review was conducted in accordance with PRISMA guidelines. A comprehensive search of PubMed, Cochrane CENTRAL, Web of Science, and Scopus was performed up to February 2025. Eligible studies included randomized controlled trials and observational cohort studies comparing liposomal bupivacaine with standard analgesic strategies in ABR. Primary outcomes were total postoperative opioid consumption and length of hospital stay. Secondary outcomes included time to ambulation, time to Foley catheter removal, time to first opioid use, and adverse events. Due to heterogeneity in study designs, interventions, and outcome reporting, a narrative synthesis was performed.

**Results:**

Ten studies (5 randomized controlled trials and 5 retrospective cohort studies) involving sample sizes ranging from 16 to 1,017 patients were included. Results for opioid consumption were heterogeneous: some studies demonstrated significant reductions with liposomal bupivacaine (e.g., 512 vs. 395 mg, *p* = 0.0001; 146.8 vs. 115.5 mg, *p* < 0.001; 79.9 vs. 40.9 mg, *p* = 0.002), while others showed no significant differences (e.g., 67 vs. 69 mg, *p* = 0.52; 215.9 vs. 211 mg, *p* = 0.883). One study reported higher opioid consumption in the liposomal bupivacaine group (240 vs. 135 mg, *p* < 0.0001). Length of hospital stay ranged from approximately 2.1 to 4.6 days, with some studies reporting modest reductions (e.g., 4.33 vs. 3.53 days, *p* = 0.0002; 3.217 vs. 2.55 days, *p* < 0.001) and others showing no difference. Time to ambulation was reduced in one study (36 vs. 21.4 h, *p* = 0.05) but not in others. Time to Foley catheter removal was shorter in one trial (2.7 vs. 2.1 days, *p* = 0.0056) but not consistently across studies. Adverse events were generally comparable between groups, although one study reported a higher rate of seroma formation with liposomal bupivacaine (11% vs. 3%).

**Conclusion:**

Liposomal bupivacaine may reduce postoperative opioid consumption in selected settings; however, its overall impact on recovery outcomes in autologous breast reconstruction remains inconsistent. Its benefits appear to depend on the underlying analgesic protocol and comparator strategy. Further high-quality, standardized randomized trials are needed to better define its role within multimodal analgesia pathways.

## 1 Introduction

Autologous breast reconstruction (ABR) has become increasingly common over the past decade owing to advances in microsurgical techniques, improved oncologic outcomes, and increased awareness of reconstructive options. National database studies have reported substantial growth in the use of autologous reconstruction, with rates more than doubling in some cohorts (1). A study investigating data from 2009 to 2016 indicated a 112% increase in ABR rates, with the overall rate growing from 26.6 to 56.5% (2). Compared with implant-based reconstruction, autologous techniques are associated with improved patient-reported outcomes, including higher satisfaction and better psychosocial and sexual well-being (3). Despite these advantages, ABR particularly abdominally based free flaps such as the deep inferior epigastric perforator (DIEP) flap is associated with significant postoperative pain originating from the abdominal donor site (4).

Acute postoperative pain following reconstruction is a common surgical outcome that typically resolves with time and appropriate pain management interventions. Inadequate pain control may delay mobilization, prolong hospital stay, and increase reliance on systemic opioids (5).

The optimal pain control profile is achieved through the use of thoracic epidural anesthesia in conjunction with intravenous patient-controlled anesthesia, transitioning to oral pain medications once the patient can tolerate oral intake (6).

Opioids have historically been the cornerstone of postoperative analgesia; however, their use is associated with adverse effects such as nausea, constipation, respiratory depression, and risk of dependence (7). These concerns have driven the adoption of multimodal, opioid-sparing analgesic strategies in reconstructive surgery (8). Regional techniques such as thoracic epidural analgesia and transversus abdominis plane (TAP) blocks are commonly used to improve pain control while reducing systemic opioid exposure (9). A study demonstrated that ultrasound-guided TAP blocks using ropivacaine decreased morphine consumption at 24 and 48 h post-procedure (10). Liposomal bupivacaine is an extended-release formulation designed to provide local analgesia for up to 72 h after administration (11). It has been investigated in several surgical fields as part of multimodal analgesia protocols, with mixed evidence regarding its effect on opioid consumption and recovery outcomes (12–14). Given the increasing use of liposomal bupivacaine in ABR and the variability in reported outcomes, a systematic synthesis of available evidence is warranted. Therefore, this systematic review aims to evaluate the effectiveness and safety of liposomal bupivacaine in patients undergoing ABR, focusing on postoperative opioid consumption, recovery outcomes, and complications.

## 2 Methods

### 2.1 Protocol and registration

We conducted this systematic review according to PRISMA checklist for systematic reviews (15) and under clear guidance of the Cochrane Handbook of Systematic Reviews and Meta-analysis of Interventions (version 5.1.0) (16).

### 2.2 Eligibility criteria

#### 2.2.1 Inclusion criteria

The inclusion criteria for this systematic review were meticulously crafted to guarantee the selection of high-caliber studies that align with the research objectives and yield dependable evidence. Eligible studies had to fulfill the subsequent PICOS criteria:

i. *Population*: Patients undergoing autologous breast reconstruction, primarily using deep inferior epigastric perforator (DIEP) flap or other abdominally based free flaps.
ii. *Intervention*: Administration of liposomal bupivacaine, either as a transversus abdominis plane (TAP) block or as part of a multimodal pain management regimen.
iii. *Comparison*: Conventional pain management strategies
iv. *Outcomes*: We included studies including one of the relevant outcomes of interest focusing of performance in different outcomes:

Primary outcomes

- Total postoperative opioid consumption (at day zero, at day one, at day two and at day three). Where possible, opioid consumption was interpreted in morphine milligram equivalents (MME); however, due to inconsistent reporting across studies, standardization was not feasible for all datasets.
- Length of hospital stay.

Secondary outcomes

- Recovery results including.

 o Time until removal of Foley catheter (days).
 o Time to ambulation

v. *Study design*: Randomized controlled trials or cohort studies.

#### 2.2.2 Exclusion criteria

The subsequent studies were excluded:

i. Study designs such as case reports, case series, reviews, and editorials.
ii. Research that did not directly evaluate the effects of liposomal bupivacaine, such as studies assessing other analgesic techniques without a direct comparison to liposomal bupivacaine.
iii. Studies that failed to report pertinent efficacy outcomes, including postoperative pain scores, opioid consumption, or patient satisfaction.
iv. Research disseminated in languages other than English due to resource limitations for translation and to avoid bias.
v. Grey literature, including conference abstracts, preprints, and non-peer-reviewed sources, due to concerns regarding incomplete reporting and methodological quality.

### 2.3 Information sources and search strategy

A literature search was conducted across four databases (PubMed, Cochrane Central Register of Controlled Trials (CENTRAL), Web of Science, and Scopus) and clinical registry databases for published articles or registries with published results up to February 2025 utilizing the following search strategy: (“liposomal bupivacaine” OR “liposome bupivacaine” OR “EXPAREL”) AND (“breast reconstruction” OR “mammoplasty” OR breast) with slight modifications for each database. Our search exclusively examined research articles published in the English language. Full search strategy for each database is provided in the Supplementary Table S1.

### 2.4 Study selection and data extraction

To prevent duplicative research, data from each database were imported into Endnote software (17) and subsequently uploaded to the Rayyan online platform (18). After being uploaded into Rayyan, records were manually reassessed to detect duplicates. The titles and abstracts of the selected studies were assessed. Subsequently, extensive texts were evaluated and assessed according to established inclusion and exclusion criteria.

Upon identifying pertinent studies that satisfy our criteria, relevant data from the included studies (such as publication year, study location, intervention and comparator group details) in addition to patients’ characteristics were extracted into standardized Google Sheet.

### 2.5 Assessment of risk of bias

We utilized the revised Cochrane risk-of-bias instrument for randomized trials (ROB 2) (19) to evaluate the potential for bias in the included clinical trials. The tool is structured according to distinct domains of bias, each focusing on various facets of trial design, execution, and documentation. Each domain employs a unique array of ‘signaling questions’ to collect information regarding trial characteristics relevant to the likelihood of bias. An algorithm employs the answers to these questions to provide a suggested evaluation of the probability of bias in each area. This evaluation may be classified as ‘Low’ or ‘High’ risk of bias, or it may reflect ‘Some concerns’. Moreover, prospective cohort studies quality was assessed using Newcastle Ottawa scale (NOS) (20). This assessment aimed to comprehensively identify potential biases that could influence the accuracy and consistency of the included studies.

## 3 Results

### 3.1 Study selection

**Figure 1:**
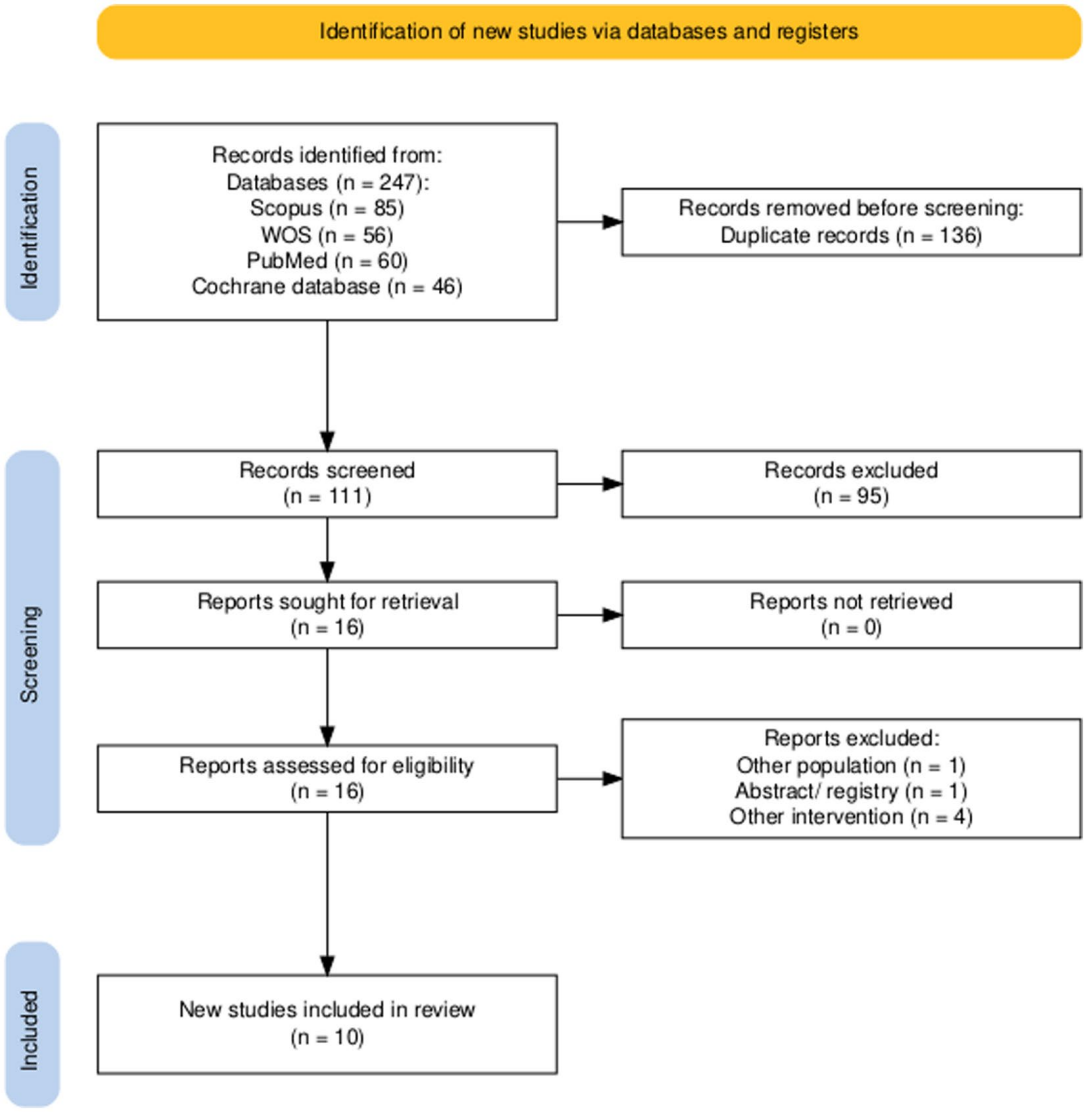
PRISMA flow diagram of selection process.

After conducting a comprehensive literature review across databases such as PubMed, Scopus, Cochrane Central, and Web of Science, we identified 247 relevant records. The utilization of Endnote and manual identification led to the elimination of 136 duplicate records. The next step involves carefully examining the remaining 111 records to verify adherence to the selection criteria. Following a thorough review of titles and abstracts, we narrowed the results to 16 articles that met our inclusion and exclusion criteria. After thorough analysis of the entire text, six records were discarded according to established criteria. The result was the identification of 10 studies (4, 7, 21–28) that were incorporated for qualitative synthesis. In addition, the reference lists of these research studies were carefully examined to uncover any pertinent publications that may have been overlooked initially. Figure 1 depicts the search approach and the quantity of papers that were included and excluded.

### 3.2 Characteristics of the studies

The systematic review included 10 studies which comprised five randomized controlled trials (RCTs) and five retrospective cohort studies, all of which were done in the United States. Each study measured the outcomes of liposomal bupivacaine use in autologous breast reconstruction surgeries with emphasis on deep inferior epigastric (DIEP) flap procedures. The intervention was liposomal bupivacaine delivery within a transversus abdominis plane (TAP) block or part of a multimodal pain control regimen. The control groups were provided with standard analgesia, which consisted of thoracic epidural anesthesia and bupivacaine, local anesthetic cocktails, or bupivacaine via continuous local anesthetic catheters. The sample size for each of the studies varied from 16 participants to 1,017. The mean age of patients included in the studies was early 50s and their BMI was in a range of 26 to 31 kg/m^2. Table 1 provides an extensive synopsis of the studies that are mentioned and baseline characteristics of patients.

**Table 1:** Summary and baseline characteristics of included studies.

Study ID	Clary, 2020 (7)		Gatherwright, 2018 (21)	Ha, 2019 (22)	Haddock, 2022 (23)	Jablonka, 2017 (24)	Knackstedt, 2024 (25)	Lombana, 2022 (26)	Nguyen, 2023 (4)	Park, 2024 (27)	Rendon, 2022 (28)
Study design	Double-blind, randomized parallel-group, clinical trial		Double-blind, randomized parallel-group, clinical trial	A Prospective, Single-Blind, Randomized, Controlled Trial	Retrospective cohort study	Retrospective cohort study	Retrospective cohort study	Retrospective cohort study	Double-blinded randomized controlled trial	Prospective single-center, single-blinded, randomized controlled trial	Retrospective cohort study
Country	US		US	US	US	US	US	US	US	US	US
Included Population	Patients who underwent deep inferior epigastric artery perforator flap–based breast reconstruction		Patients undergoing delayed, unilateral DIEP reconstruction for breast cancer	Patients undergoing abdominally based autologous breast reconstruction	Patients undergoing DIEP flap breast reconstruction.	Patients who underwent breast reconstruction using abdominally based free flaps.	Patients undergoing primary DIEP flap breast reconstruction.	Patients who underwent abdominally based autologous breast reconstruction.	Patients undergoing abdominally based autologous breast reconstruction.	Patients undergoing DIEP free flap breast reconstruction	Patients who underwent autologous breast reconstruction
Intervention (I)	Transversus Abdominis Plane Block With Liposomal Bupivacaine		266 mg of liposomal bupivacaine diluted in 60 mL along with 20 cc ¼% bupivacaine	266 mg of liposomal bupivacaine	ERAS plus liposomal bupivacaine group	A nonnarcotic pain control regimen plus liposomal bupivacaine	Liposomal bupivacaine	Liposomal bupivacaine	Liposomal bupivacaine	Liposomal bupivacaine	Single-shot of liposomal bupivacaine
Control (C)	Thoracic epidural anesthesia		0.25% of bupivacaine	75 mg of conventional bupivacaine	ERAS group	A nonnarcotic pain control regimen	Bupivacaine	No liposomal bupivacaine	Bupivacaine	Bupivacaine / epinephrine	Continuous local anesthetic catheter
Sample Size, *N*	I	15	8	22	80	40	669	38	30	58	39
	C	15	8	22	69	48	348	36	30	59	60
Age (yrs), mean (SD)	I		52.1 (NR)	49 (9.2)	52 (9.2)	50.2 (8.5)	51.7 (9.8)	48.6 (9.8)	53 (9.5)	51.1 (8.7)	54.9 (8.9)
	C		53.1 (NR)	49 (10)	52.1 (9.2)	50.6 (8.8)	50.4 (9.2)	51 (9)	52.2 (9.8)	51.9 (10.5)	50.3 (10.4)
BMI (Kg), mean (SD)	I		30 (NR)	29.1 (4.6)	30.1 (4.8)	28 (5.4)		30.5 (5.1)	29.6 (5.3)	30 (6.3)	31.3 (5)
	C		28 (NR)	28.1 (4.5)	30.6 (5)	26.2 (5)		30.2 (4.7)	30.2 (4.3)	31.6 (6.7)	30.1 (5.7)
Smoking (Current or former), *N* (%)	I				22 (27.5)				1 (3)	18 (30.5)	13 (33)
	C				15 (21.7)				1 (3)	18 (32.2)	18 (30)
Unilateral reconstruction, *N* (%)	I	7 (47)	8 (100)	8 (36.4)	N/A	18 (45)	295 (44.1)	8 (21)	9 (30)	30 (50.8)	19 (49)
	C	5 (33)	8 (100)	8 (36.4)	N/A	30 (62.5)	93 (26.7)	9 (25)	7 (23)	25 (42.4)	38 (63)
Race (White), mean (SD)	I			17 (77)	56 (70)						
	C			22 (100)	47 (68.1)						
Hypertension, *N* (%)	I			3 (13.6)	23 (28.8)			12 (32)	9 (31)		15 (39)
	C			2 (9.1)	18 (26.1)			17 (47)	10 (34)		15 (25)
Diabetes, *N* (%)	I			1 (4.5)	9 (11.3)			5 (13)	8 (27)	5 (8.6)	4 (10)
	C			0 (0)	7 (10.1)			7 (19)	6 (21)	8 (13.6)	4 (7)
Radiotherapy, *N* (%)	I			12 (54.5)	20 (25)	12 (30)		27 (71)	6 (20)	16 (27.1)	
	C			15 (68.2)	34 (49.3)	14 (29.2)		19 (53)	10 (33)	13 (22)	
Chemotherapy, *N* (%)	I			17 (77.3)	24 (30)	7 (17.5)		12 (32)	10 (33)	19 (32.2)	
	C			17 (77.3)	35 (50.7)	10 (20.8)		18 (50)	10 (33)	11 (18.6)	

NR, not reported; DIEP, deep inferior epigastric perforator; ERAS, enhanced recovery after surgery.

### 3.3 Risk of bias and quality assessment

**Figure 2:**
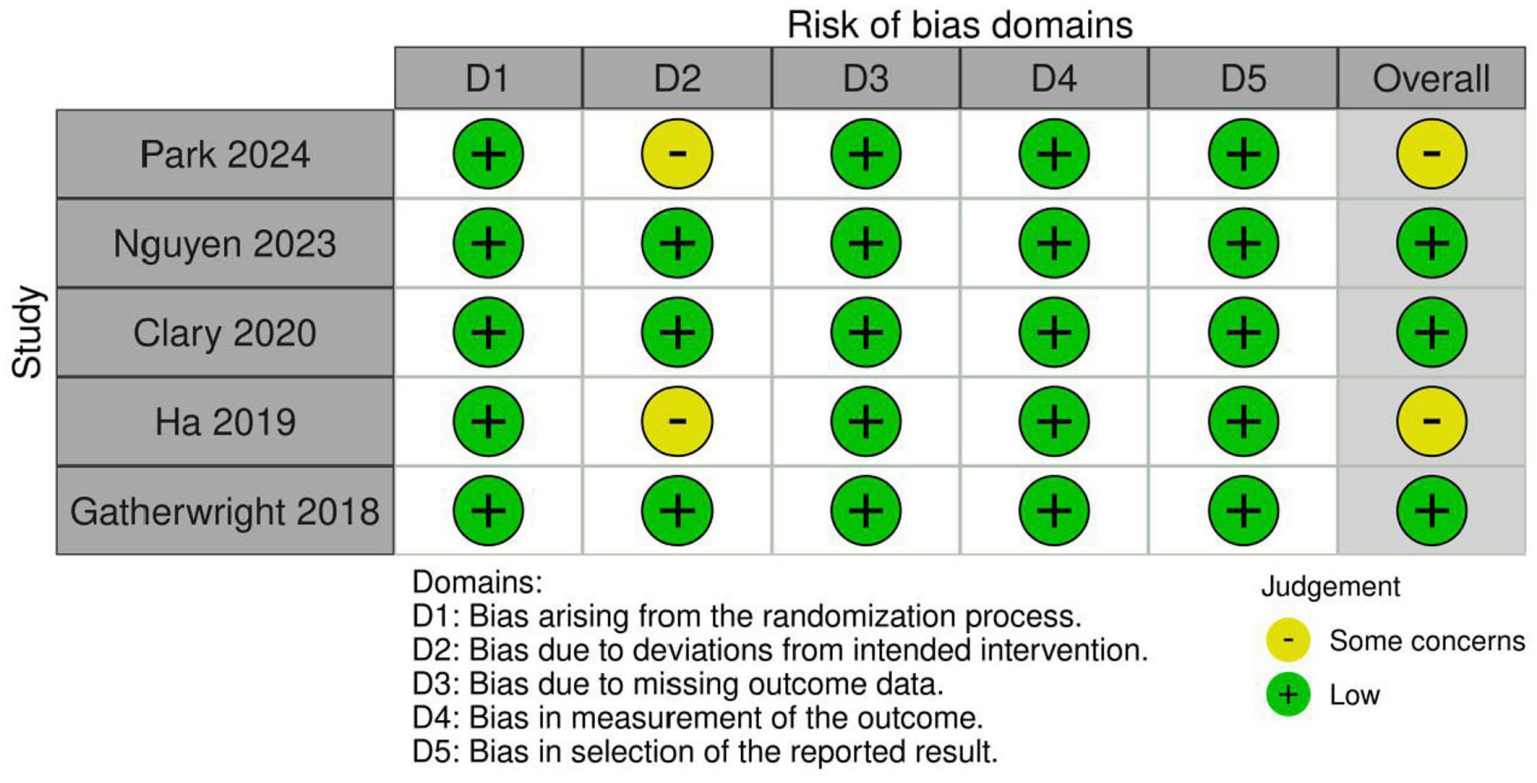
Risk of bias graph.

The risk of bias assessment for the included RCTs was performed using the ROB2 tool. The evaluation revealed that three trials showed low risk of bias with high quality of evidence and two trials showed some concerns. Figure 2 provides a detailed risk of bias summary, illustrating the judgment for each domain across all included trials. Quality assessment of cohort studies were done using NOS and revealed an overall good quality of included cohort studies as shown in Supplementary Table S2.

### 3.4 Efficacy outcomes

#### 3.4.1 Primary outcome

##### 3.4.1.1 Opioid consumption

Total postoperative opioid consumption differs in studies analyzing liposomal bupivacaine versus a standard comparator. Some studies demonstrated a significant reduction in opioid consumption with liposomal bupivacaine while others showed no significant difference. Clary (7) showed no significant difference in total postoperative opioid consumption by group (253.1 vs. 205.4, *p* = 0.2743), however, there was significantly lower opioid consumption on postoperative day 3 in the liposomal bupivacaine group (59.6 vs. 24.5, *p* = 0.005). Haddock (23) showed total opioid consumption to be significantly less in the liposomal bupivacaine group (146.8 vs. 115.5, *p* < 0.001). Knackstedt (25) reported lower opioid use during the perioperative time with liposomal bupivacaine (512 vs. 395, *p* = 0.0001) and during the inpatient period (303 vs. 154, *p* < 0.0001). Lombana (26) reported liposomal bupivacaine resulted in significantly less opioid consumption (240 vs. 633, *p* < 0.0001). Other studies like Nguyen (4) and Rendon (28) failed to find significant differences in opioid use between groups (*p* = 0.52 and *p* = 0.883 respectively).

#### 3.4.2 Secondary outcomes

##### 3.4.2.1 Duration of hospital stay

The impact of liposomal bupivacaine on the duration of hospital stays was somewhat inconsistent:

Clary (7) and Haddock (23) reported significant decreases in the length of stay associated with liposomal bupivacaine (4.33 vs. 3.53 days, *p* = 0.0002; 3.217 vs. 2.55 days, *p* < 0.001, respectively). Jablonka (24) noted a significant reduction (3.52 vs. 2.65 days, *p* < 0.001), as did Gatherwright (21) (3.63 vs. 3.38, *p* = 0.08, which was not significant). Knackstedt (25) and Lombana (26) found no significant differences between groups (*p* = 0.334 and *p* = 0.1, respectively). Park (27) and Rendon (28) also found no significant difference *p* = 0.55 and *p* = 0.953, respectively.

##### 3.4.2.2 Time to ambulation

Gatherwright (21) noted that the time to ambulation was significantly shorter in the liposomal bupivacaine group (36 vs. 21.43 h, *p* = 0.05). Ha (22) did not report a significant difference (2.2 vs. 1.81 days, *p* = 0.64).

##### 3.4.2.3 Time to first opioid use

Nguyen (4) did not find a significant difference between groups (8.2 vs. 12.4 h, *p* = 0.41).

##### 3.4.2.4 Time until Foley catheter removal

Clary (7) noted a significant decrease in catheter duration with liposomal bupivacaine (2.7 vs. 2.1 days, *p* = 0.0056). Ha (22) did not find a significant difference (1.27 vs. 1.02 days, *p* = 0.38).

Detailed information about outcomes is represented in Table 2.

**Table 2:** Outcomes findings of included studies.

Study and outcomes assessed	Comparator	Liposomal bupivacaine	*p* value
Clary, 2020 (7)			
Total postoperative opioid consumption (Day 0)	34.9	32.6	0.81
Total postoperative opioid consumption (Day 1)	98.9	92.4	0.78
Total postoperative opioid consumption (Day 2)	59.7	56	0.79
Total postoperative opioid consumption (Day 3)	59.6	24.5	0.005
Total postoperative opioid consumption	253.1	205.4	0.2743
Time until removal of Foley catheter (days)	2.7	2.1	0.0056
Length of stay	4.33	3.53	0.0002
Gatherwright, 2018 (21)			
Length of stay	3.63	3.38	0.08
Average total opioid utilization	79.9	40.9	0.002
Time to ambulation	36	21.43	0.05
Ha, 2019 (22)			
Time until removal of Foley catheter (days)	1.27	1.02	0.38
Time to ambulation	2.2	1.81	0.64
Total postoperative opioid consumption	300	283	0.98
Haddock, 2022 (23)			
Total postoperative opioid consumption	146.8	115.5	<0.001
Length of stay	3.217	2.55	<0.001
Jablonka, 2017 (24)			
Length of stay	3.52	2.65	<0.001
Total postoperative opioid consumption (Day 1)	7.25	1.88	0.0081
Total postoperative opioid consumption (Day 2)	5.17	2.12	0.027
Total postoperative opioid consumption (Day 3)	3.6	2.23	0.215
Knackstedt, 2024 (25)			
Opioid intake during perioperative period	512	395	0.0001
Opioid intake 72 h after surgery	140	63	<0.0001
Opioid intake during the entire inpatient period	303	154	<0.0001
Length of stay	4.6	4.5	0.334
Lombana, 2022 (26)			
Total postoperative opioid consumption	633	240	<0.0001
Length of stay	4.7	4.1	0.1
Nguyen, 2023 (4)			
Total postoperative opioid consumption	67	69	0.52
Length of stay	2.42	2.23	0.2
Time to first opioid use	8.2	12.4	0.41
Park, 2024 (27)			
Total postoperative opioid consumption (Day 1)	40	33.8	0.42
Total postoperative opioid consumption (Day 2)	22.5	15	0.69
Total postoperative opioid consumption	55	50	0.47
Length of stay	2.1	2.2	0.55
Rendon, 2022 (28)			
Total postoperative opioid consumption	215.9	211	0.883
Length of stay	3	3	0.953

### 3.5 Safety outcomes

#### 3.5.1 Adverse events

Adverse events were different in different studies, but some indications were noted:

Clary (7) found no difference in rates of pruritus (33% for both groups), but lower rates of constipation (38.5% vs. 46.7%), nausea (33% vs. 66%), and emesis (7.1% vs. 13.3%) were noted with liposomal bupivacaine. Jablonka (24) noted liposomal bupivacaine had lower major complications (7.5% vs. 12.5%), 30-day readmission (2.5% vs. 6.25%), return to the operating room (7.5% vs. 14.5%), and transfusion (2.5% vs. 23%) rates.

Lombana (26) observed similar rates of surgical site infection (21% vs. 20%), while hematoma was higher in the comparator group 0% vs. 10%. Liposomal bupivacaine was more frequent, however, in cases of seroma (11% vs. 3%).

Park (27) described similar rates of wound dehiscence (12.1% vs. 11.9%), wound infection (0% vs. 1.7%), hospital readmission (3.4% vs. 1.7%), hematoma (3.4% vs. 3.4%) and flap failure (1.7% vs. 1.7%).

Detailed information about safety outcomes is illustrated in Table 3.

**Table 3:** Complications.

Adverse events	Liposomal bupivacaine	Comparator
Clary, 2020 (7)		
Pruritus	33%	33%
Constipation	38.5%	46.7%
Nausea	33%	66%
Emesis	7.1%	13.3%
Jablonka, 2017 (24)		
Total major	7.5%	12.5%
30-day readmission	2.5%	6.25%
Return to OR	7.5%	14.5%
Flap loss	5%	4.17%
Transfusions	2.5%	23%
Lombana, 2022 (26)		
Surgical-site infection	21%	20%
Hematoma	0%	10%
Seroma	11%	3%
Hospital readmission	11%	10%
Return to OR	13%	17%
Park, 2024 (27)		
Wound dehiscence	12.1%	11.9%
Wound infection	0%	1.7%
Hospital readmission	3.4%	1.7%
Hematoma	3.4%	3.4%
Seroma	0%	1.7%
Flap failure	1.7%	1.7%

## 4 Discussion

To optimize recovery, reduce complications, and increase patient satisfaction, effective pain management is crucial (29). Opioid analgesics have historically dominated postoperative pain control; however, the widespread use of opioids can lead to adverse outcomes such as respiratory depression, nausea, constipation, and dependence (23). This has led to increased interest in non-opioid alternatives, which serve as an adjunct in multimodal analgesia strategies (30). Liposomal bupivacaine, an extended-release formulation, has been used in some circumstances because it enables prolonged regional analgesia without escalating the use of systemic opioids. In this discussion, the effects of liposomal bupivacaine on opioid consumption, length of stay, and time to ambulatory activity postoperatively are analyzed (31).

### 4.1 Main findings

Liposomal bupivacaine has had a varying effect on opioid consumption after surgery. Some pieces of research suggest that patients utilized fewer opioids, likely owing to its prolonged release mechanism that provides analgesia over time and reduces the need for systemic opioids. However, the differences in surgical procedures, dosing strategies, and patient-related variables are major factors why some studies present contradicting results. Liposomal bupivacaine may be ineffective alone in high postoperative pain surgeries and require the assistance of opioid analgesics. The impact of liposomal bupivacaine on the length of hospital stays, however, is questionable. Some studies report a decrease in length, which may be attributed to better pain control allowing early mobilization and lessened side effects from opioids. Other institutional factors such as protocol and surgical complexity may, however, cloud the benefits offered by better analgesia. Just like with the length of stay, the effect on the time to ambulation is mixed. While some studies note earlier mobility due to pain relief, others find no significant difference, pointing toward additional institutional and patient-related factors. With respect to safety outcomes, reductions in opioid-related adverse effects may be observed, potentially attributable to decreased opioid use and improved hemodynamic stability. However, these benefits raise concern regarding increased risks of seromas and other postoperative wound complications, highlighting the need for stringent patient selection and careful surgical strategy.

### 4.2 Investigation with prior literature

The findings of this review are consistent with prior systematic reviews across surgical specialties, which have reported mixed or procedure-dependent benefits of liposomal bupivacaine. A systematic review by Vyas et al. (13) found that although liposomal bupivacaine may reduce postoperative pain scores and opioid use in some plastic surgery procedures, the evidence was heterogeneous and often limited by small sample sizes and varying analgesic protocols.

Similarly, Hamilton et al. (32) conducted a meta-analysis across multiple surgical fields and concluded that liposomal bupivacaine did not consistently demonstrate clinically meaningful improvements over standard local anesthetics, particularly when used within established multimodal analgesia pathways.

In the context of abdominally based autologous breast reconstruction, ERAS protocols have been shown to reduce opioid consumption and hospital stay through multimodal analgesia strategies, including regional anesthesia techniques such as TAP blocks or epidural analgesia. In such optimized pathways, the incremental benefit of a single analgesic intervention, including liposomal bupivacaine, may be less pronounced. This may explain the variability observed among the included studies, where institutional protocols and comparator analgesic regimens differed substantially (33, 34).

### 4.3 Clinical implications

The findings of this systematic review suggest that liposomal bupivacaine may have a role as an adjunct within multimodal analgesia protocols for ABR, particularly in abdominally based free flap procedures. Several included studies demonstrated reductions in postoperative opioid consumption, which is clinically relevant given the well-documented adverse effects of opioids, including nausea, ileus, sedation, respiratory depression, and the risk of persistent opioid use after surgery (35).

Current perioperative pain management guidelines emphasize the use of multimodal, opioid-sparing strategies to improve recovery and reduce complications. The American Pain Society and other consensus guidelines recommend combining regional anesthesia, non-opioid systemic analgesics, and local anesthetic techniques to optimize postoperative outcomes (35, 36). In microsurgical breast reconstruction, ERAS protocols incorporating multimodal analgesia have been associated with reduced opioid consumption, earlier mobilization, and shorter hospital stays (33, 37).

Within this context, liposomal bupivacaine may offer advantages because of its extended-release formulation, which can provide local analgesia for up to 72 h (38). This duration aligns with the period of peak postoperative pain following abdominal donor-site surgery, suggesting a potential role in reducing early opioid requirements. Some studies in this review demonstrated reductions in total opioid consumption and, in certain cases, shorter hospital stays when liposomal bupivacaine was incorporated into perioperative protocols.

However, the clinical benefit appears to be influenced by the underlying analgesic pathway. In studies where liposomal bupivacaine was compared with less comprehensive or opioid-based regimens, reductions in opioid use were more apparent. In contrast, when it was compared with optimized ERAS protocols or other regional techniques such as thoracic epidural or continuous local anesthetic infusion, the incremental benefit was less consistent. This observation is supported by prior systematic reviews, which have shown that liposomal bupivacaine does not consistently outperform standard local anesthetics when used within structured multimodal pathways (31, 32).

Therefore, liposomal bupivacaine should be viewed as a potential component of a comprehensive analgesic strategy rather than a standalone solution. Its use may be particularly relevant in settings where: Epidural analgesia is contraindicated or not routinely used, institutional protocols favor TAP blocks or local infiltration techniques, patients are at higher risk for opioid-related complications, and early mobilization and discharge are key priorities.

Nevertheless, cost considerations and institutional resource availability must also be taken into account. Liposomal bupivacaine is substantially more expensive than conventional local anesthetics, and cost-effectiveness analyses have produced mixed results across surgical specialties (39). As a result, its routine use should be guided by institutional protocols, patient selection, and economic considerations.

### 4.4 Limitations

There are several restrictions that need to be accepted even with the most optimistic results. The differences in the designs of the studies, the performed surgeries, and the available patients introduce heterogeneity that may influence the generalizability of the results. Furthermore, the differences in the dosing interval and the protocols for multimodal analgesia make comparison between the studies more difficult. Also, some studies had insufficient follow up for chronic cases which made studying the long-term benefits or possible chronic complications of liposomal bupivacaine impossible. These findings suggest that there is a need for randomized clinical trials of greater methodological rigor which will test the newer form’s application in pain management general surgery.

## 5 Conclusion

The outcomes of this systematic review imply that liposomal bupivacaine may improve specific recovery outcomes and reduce opioid utilization. Its analgesic effect duration is consistent with its purported mechanism, however, its effect on length of stay, ambulation, and other recovery outcomes are inconsistent. These differences are likely due to institutional protocols, variability among patients, and the procedure itself. Even though there seems to be a positive aspect to the safety profile in regard to opioid complications, further investigation is required to determine its influence on wound healing and post-operative complications. Liposomal bupivacaine continues to be a candidate for an adjunct in multidrug analgesia regimens, but his may be most evident in certain surgical procedures and patient groups.

## Data Availability

The original contributions presented in the study are included in the article/Supplementary material, further inquiries can be directed to the corresponding author.
