# Supplementary material for: Effectiveness and safety of liposomal bupivacaine in autologous breast reconstruction: a systematic review
Source: Front Med (Lausanne). 2026 May 7;13:1805974. doi: 10.3389/fmed.2026.1805974 (PMC13190593; doi:10.3389/fmed.2026.1805974)
Supplement: Supplementary file 1 [file Supplementary_file_1.docx]

Table S1: Search strategy for each database

| **Database** | **Search Filter / Field** | **Full Search Strategy** |
| --- | --- | --- |
| **PubMed** | Title/Abstract | ("liposomal bupivacaine"[Title/Abstract] OR "liposome bupivacaine"[Title/Abstract] OR "EXPAREL"[Title/Abstract]) AND ("breast reconstruction"[Title/Abstract] OR "autologous breast reconstruction"[Title/Abstract] OR "DIEP"[Title/Abstract] OR "deep inferior epigastric perforator"[Title/Abstract] OR "mammoplasty"[Title/Abstract] OR "breast surgery"[Title/Abstract]) |
| **Cochrane CENTRAL** | Title/Abstract | ("liposomal bupivacaine" OR "liposome bupivacaine" OR "EXPAREL") AND ("breast reconstruction" OR "autologous breast reconstruction" OR "DIEP" OR "deep inferior epigastric perforator" OR "mammoplasty" OR "breast surgery") |
| **Scopus** | Abstract only | (ABS("liposomal bupivacaine") OR ABS("liposome bupivacaine") OR ABS("EXPAREL")) AND (ABS("breast reconstruction") OR ABS("autologous breast reconstruction") OR ABS("DIEP") OR ABS("deep inferior epigastric perforator") OR ABS("mammoplasty") OR ABS("breast surgery")) |
| **Web of Science** | Title, Abstract, Keywords | TS=("liposomal bupivacaine" OR "liposome bupivacaine" OR "EXPAREL") AND TS=("breast reconstruction" OR "autologous breast reconstruction" OR "DIEP" OR "deep inferior epigastric perforator" OR "mammoplasty" OR "breast surgery") |

Table S2 showing quality assessment of cohort studies using NOS

| **Study ID** | **Selection** | | | | **Comparability** | **Outcome** | | | **Overall Score** |
| --- | --- | --- | --- | --- | --- | --- | --- | --- | --- |
|  | **Representativeness of the exposed cohort** | **Selection of the non-exposed cohort** | **Ascertainment of exposure** | **Demonstration that outcome of interest was not present at start of study** | **Comparability of cohorts on the basis of the design or analysis** | **Assessment of outcome** | **Was follow-up long enough for outcomes to occur** | **Adequacy of follow up of cohorts** |  |
| **Haddock 2022** | * | * | * | * | ** | * | * | * | 9 |
| **Jablonka 2017** | * | * | * | * | ** | * | * | * | 9 |
| **Knackstedt 2024** | * | * | * | * | ** | * | * | * | 9 |
| **Lombana 2022** | * | * | * | * | ** | * | * | * | 9 |
| **Rendon 2022** | * | * | * | * | * | * | * | * | 8 |
